# Novel Pressure Wave Separation Analysis for Cardiovascular Function Assessment Highlights Major Role of Aortic Root

**DOI:** 10.1109/TBME.2021.3127799

**Published:** 2022-04-21

**Authors:** Samuel Vennin, Ye Li, Jorge Mariscal-Harana, Peter H. Charlton, Henry Fok, Haotian Gu, Phil Chowienczyk, Jordi Alastruey

**Affiliations:** King’s College London British Heart Foundation, Department of Clinical Pharmacology, St Thomas’ Hospital, U.K., School of Biomedical Engineering and Imaging Sciences, King’s College London, St Thomas’ Hospital, U.K.; King’s College London British Heart Foundation, Department of Clinical Pharmacology, St Thomas’ Hospital, U.K.; School of Biomedical Engineering and Imaging Sciences, King’s College London, St Thomas’ Hospital, U.K.; School of Biomedical Engineering and Imaging Sciences, King’s College London, St Thomas’ Hospital, U.K.; King’s College London British Heart Foundation, Department of Clinical Pharmacology, St Thomas’ Hospital, U.K.; King’s College London British Heart Foundation, Department of Clinical Pharmacology, St Thomas’ Hospital, U.K.; King’s College London British Heart Foundation, Department of Clinical Pharmacology, St Thomas’ Hospital, U.K.; School of Biomedical Engineering and Imaging Sciences, King’s College London, St Thomas’ Hospital, London SE1 7EH, U.K.

**Keywords:** Cardiovascular mechanics, hypertension, pulse wave reflections, aortic root, arterial blood pressure

## Abstract

**Objective:**

A novel method was presented to separate the central blood pressure wave (CBPW) into five components with different biophysical and temporal origins. It includes a time-varying emission coefficient (γ) that quantifies pulse wave generation and reflection at the aortic root.

**Methods:**

The method was applied to normotensive subjects with modulated physiology by inotropic/vasoactive drugs (n = 13), hypertensive subjects (n = 158), and virtual subjects (n = 4,374).

**Results:**

*γ* is directly proportional to aortic flow throughout the cardiac cycle. Mean peak *γ* increased with increasing pulse pressure (from <30 to >70 mmHg) in the hypertensive (from 1.6 to 2.5, P < 0.001) and *in silico* (from 1.4 to 2.8, P < 0.001) groups, dobutamine dose (from baseline to 7.5 *μ*g/kg/min) in the normotensive group (from 2.1 to 2.7, P < 0.05), and remained unchanged when peripheral wave reflections were suppressed *in silico.* This was accompanied by an increase in the percentage contribution of the cardiac-aortic-coupling component of CBPW in systole: from 11% to 23% (P < 0.001) in the hypertensive group, 9% to 21% (P < 0.001) in the *in silico* group, and 17% to 23% (P < 0.01) in the normotensive group.

**Conclusion:**

These results suggest that the aortic root is a major reflection site in the systemic arterial network and ventricular-aortic coupling is the main determinant in the elevation of pulsatile pulse pressure.

**Significance:**

Ventricular-aortic coupling is a prime therapeutic target for preventing/treating systolic hypertension.

## I Introduction

REFLECTED waves are considered to be a major contributor to the central (aortic) blood pressure wave (CBPW), especially in determining the augmentation pressure; *i.e.*, the pressure buildup from the first systolic peak or shoulder to the second systolic peak which in middle-aged to older subjects usually corresponds to peak pressure [1]. For this reason, reflected waves have historically been associated with elevated blood pressure (BP) [2], although recent studies have also emphasized the importance of ventricular ejection patterns in shaping the BP wave in the first half of systole [3], [4]. Furthermore, it is now commonly accepted that there is a myriad of reflection sites in the arterial tree [5], [6] – any site of impedance mismatch can generate reflected waves – and early models used to justify the presence of a main reflection site [7], [8] are now considered too simplistic [6], [9].

**Fig. 1:**
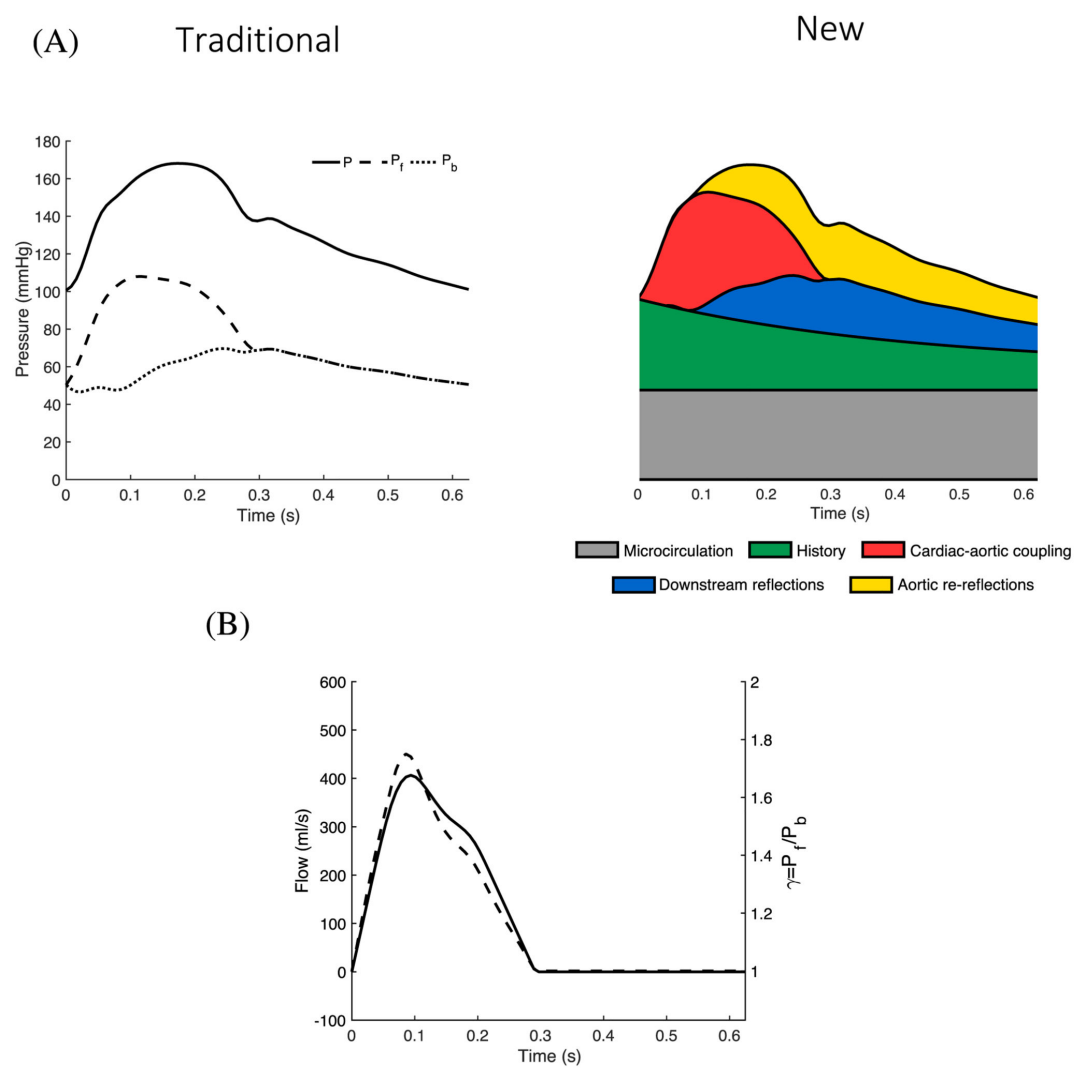
(A) Comparison between (left) traditional decomposition of central blood pressure into forward (*P*_f_, dashed) and backward (*P*_b_, dotted) waves and (right) our new decomposition into five components with different biophysical and temporal origins: (i) microcirculation (grey), history (green), cardiac-aortic coupling (red), downstream reflections (blue) and aortic re-reflections (yellow). (B) Relation of emission coefficient, *γ* (dashed), computed as the ratio of *P*_f_ to *P*_b_, to aortic flow wave (solid).

Traditional wave separation analysis (WSA) can provide insights into the direction of the pulsatile components of CBPW: forward traveling from the heart to the periphery or backward traveling from the periphery to the heart. However, it can only identify the origin (distal or proximal) of the waves that make up CBPW at a given time (Fig. 1(A)) and does not provide information about the physical locations in the cardiovascular system where those waves originated [10]. For example, the forward-traveling wave in systole is composed in part of the wave produced by the coupling of ventricular flow ejection with aortic impedance (the so-called water hammer pressure). The water hammer pressure propagates from the heart to the periphery in agreement with the ‘forward’ WSA terminology. However, there is a portion of the net forward wave that becomes increasingly important from the time of peak ejection onwards and which is made up of reflected waves that travel back to the aorta and are re-reflected at the aortic root [11]. In diastole, these re-reflected waves entirely make up the forward pressure wave as the reflection coefficient at the aortic root is close to unity [12].

In this study, we introduced a new WSA to quantify the relative contributions of cardiac and vascular properties to CBPW. The analysis separates CBPW into five well-defined components with different biophysical and temporal origins (Fig. 1(A)). Furthermore, it quantifies wave reflections at the aortic root by introducing a time-varying emission coefficient, *γ*, defined as the ratio of forward- to backward-traveling central blood pressure waves, which closely matches the aortic flow wave ejected by the left ventricle (LV) for the whole cardiac cycle (Fig. 1(B)). This new separation notably differentiates between waves arising during the current cardiac cycle and those originating from previous cycles, while highlighting the forward re-reflections of downstream reflections at the aortic root. We demonstrated the utility of our new method in three case studies involving complementary *in vivo* and *in silico* hemodynamic data, which emphasized the major role of the aortic root in shaping CBPW during systole.

## II Materials

### A *In Vivo* Data

*In vivo* data were those previously obtained in a group of hypertensive subjects (n = 158, 83 men, age 46±17 years, BP 130±28/83±21 mmHg, mean±SD) and normotensive volunteers (n = 13; 10 men; age 49±8 years; BP 110±16/65±3 mmHg, mean±SD) [13], [14]. Characteristics of the hypertensive and normotensive groups are given in Supplemental Material Tables S1 and S2, respectively. Healthy volunteers took part in crossover studies to investigate the change in pulsatile hemodynamics during administration of drugs with different inotropic and vasopressor/vasodilator properties: dobutamine (a positive inotrope with some vasodilator actions), norepinephrine (a vasoconstrictor with some inotropic actions), phentolamine (a small artery dilator), and nitroglycerin (predominantly a large artery dilator with some action on ventricular dynamics and venodilation). Each subject received at least 2 comparator drugs: either the vasopressor agent dobutamine and norepinephrine or the vasodilators phentolamine and nitroglycerin, and data for each drug were obtained on at least 10 subjects. Each drug was given on a different occasion separated by at least 7 days, and the order was randomized. Measurements were performed in a quiet temperature controlled (24-26 °C) vascular laboratory, and subjects were asked to avoid caffeine and alcohol intake on the day of the study. On arrival in the vascular laboratory, a peripheral venous catheter was inserted into the left antecubital fossa through which 0.9% saline (Baxter Healthcare) vehicle or drugs dissolved in saline were infused at 1 mL/min using a syringe driver (Injectomat; Agilia; Fresenius Kabi, Bad Homburg, Germany). After 30-minute resting supine during infusion of saline vehicle, baseline hemodynamic measurements were made as detailed below. On different occasions, dobutamine (2.5, 5, and 7.5 *μ*g/kg per minute; Hameln Pharmaceuticals, Gloucester, United Kingdom), norepinephrine (12.5, 25, and 50 ng/kg per minute; Aguettant, Bristol, United Kingdom), phentolamine (1 mg bolus+25 *μ*g/min, 2 mg+50 *μ*g/min, and 4 mg+100 *μ*g/min; Alliance Pharmaceuticals, Chippenham, United Kingdom), and nitroglycerin (3, 10, and 30 *μ*g/min; Hospira Incorporation, Lake Forest, IL) dissolved in 0.9% saline vehicle were then infused at 1 mL/min, and hemodynamic measurements were repeated at each drug dose when steady state was achieved after at least 7 minutes of infusion. In hypertensive patients, measurements were made at baseline only. The studies during which those data were collected were approved by the London Westminster Research Ethics Committee (REC reference number: 11/H0802/5; date of approval: Feb 2011), and written informed consent was obtained.

### B Hemodynamic Measurements

Hemodynamic measurements were performed as previously described [13]. Radial and carotid pressure waveforms were obtained by applanation tonometry performed by an experienced operator using the SphygmoCor system (AtCor, Australia). For each measurement, approximately 10 cardiac cycles were obtained and ensemble averaged. Waveforms that did not meet the in-built quality control criteria in the SphygmoCor system were rejected. Brachial BP was measured in triplicate by a validated oscillometric method (Omron 705CP, Omron Health Care, Japan) immediately before measurements of tonometry and used to calibrate radial waveforms, and thus to obtain a mean arterial pressure (MAP) through integration of the radial waveform. Carotid waveforms were calibrated from MAP and diastolic brachial blood pressures (DBP) on the assumption of equality between proximal and peripheral DBP [15]. Ultrasound imaging was performed by an experienced operator using a Vivid-7 ultrasound platform (General Electric Healthcare, U.K.). This provided a measurement of the flow velocity above the aortic valve using pulsed wave Doppler obtained from an apical five-chamber view. All ultrasound measurements were extracted from the envelope of the spectrum, filtered to reduce speckles in late systole and early diastole, and averaged over at least three cardiac cycles.

### C *In Silico* Data

A population of virtual subjects presenting a range of cardiovascular properties representative of a sample of healthy adults over 5 decades (from 25 to 75 year old) was used [16]. Briefly, for each virtual subject, the CBPW, *P*(*t*), was computed from a prescribed aortic flow waveform, *Q*(*t*), using a 116-artery distributed model with physical arterial properties including cross-sectional area and pulse wave velocity, *c*, specified for each arterial segment. Characteristics of the time-varying *Q*(*t*) (heart rate and stroke volume) and physical characteristics of the arterial tree (cross-sectional area, arterial and peripheral compliance, proximal aortic length, peripheral vascular resistance) were varied over the typical physiological range for each age decade to produce 4,374 virtual subjects in total. In the last case study where the impact of peripheral reflections on the emission coefficient at the aortic root was assessed (Section E), we simulated separately baseline models for each age decade with both normal and totally absorbent terminal boundary conditions, following the method described in [10]. For comparison purposes, we then combined the two simulations to replicate the scenario where terminal boundary conditions are switched from normal to totally absorbent at the end of a cardiac cycle. This was performed by adding the net pressure wave obtained in the totally absorbent case to the micro-circulation and history pressure components from the normal case.

### D Waveform Postprocessing

For the *in vivo* data, ensemble-averaged carotid pressure was used as surrogate for CBPW [17]. This pressure wave, together with the Doppler ultrasound aortic flow velocity wave and the *in silico* CBPW and aortic flow waves, were processed offline using custom software written in MATLAB (MathWorks, Natick, USA).

### E Case Studies

We applied our new WSA in three different case studies. In the first one, we investigated changes in the WSA components with increasing pulse pressure (PP) in selected groups: the *in vivo* hypertensive group and the population of virtual subjects. This was achieved by dividing both groups into 10-mmHg spans of increasing PP and calculating the ensemble-averaged CBPW for each span. The contribution of each WSA component to the ensemble-averaged CBPW of each span was then quantified in terms of percentage area occupied under the CBPW during systole. In the second case study, we used a subset of the *in vivo* normotensive group where patients were administered increasing doses of dobutamine to check the ability of the WSA to account for enhanced myocardial contractility. Variations in each WSA component with the increasing dobutamine dose were studied by performing the same analysis as described for the first case study. In the third case study, the baseline subjects for each age decade of the population of virtual subjects were used to simulate a scenario in which all terminal boundary conditions are switched at the end of the previous cardiac cycle from normal to totally absorbent. This was performed to assess the impact of peripheral wave reflections on the emission coefficient at the aortic root.

### F Statistics

Subject characteristics and results are presented as mean±SD. For the case studies, comparisons of subject characteristics across groups were made by one-way ANOVA. Depending on the sample size, statistical significance was either set at P < 0.001 (first case study with a large number of samples, n = 158) or P < 0.05 (second case study with a small number, n = 10). Analysis was performed using SPSS version 22.0 (SPSS Inc. Chicago, Illinois).

## III Methods

### A Traditional Pressure Wave Separation

The traditional decomposition of CBPW was performed using Parker’s time-domain approach [18] to obtain the forward, *P_f_*(*t*), and backward, *P_b_*(*t*), traveling components of CBPW with time *t* (Fig. 1(A)) from the net pressure, *P*(*t*), flow velocity, *U*(*t*), blood density, *ρ*, diastolic blood pressure, *DBP*, and pulse wave velocity, *c*. These are given by [19]

(1) $$\begin{array}{l} P_{f}=\frac{1}{2}\sum \left(dP+\rho cdU\right)+\frac{DBP}{2},\\ P_{b}=\frac{1}{2}\sum \left(dP-\rho cdU\right)+\frac{DBP}{2} \end{array}$$

Both *P_f_* and *P_b_* have the same value DBP/2 at pressure onset (so that *P = P_f_ + P_b_*) under the assumption that, in late diastole, *P_b_* is reflecting against a closed aortic valve with a reflection coefficient equal to unity [20]. Pulse wave velocity was calculated using the sum-of-squares method [21], both for the *in vivo* and *in silico* data.

### B Emission Coefficient *γ* at The Aortic Root

Reflection coefficients in the arterial system are often computed as the ratio of the peak magnitudes of outgoing to incoming waves, with incoming waves traveling forward; *i.e.*, towards the downstream vasculature. At the aortic root, the incoming wave is the traditional backward-traveling wave, *P_b_*(*t*), while the outgoing wave is the forward-traveling wave, *P_f_*(*t*), which is composed of re-reflections of *P_b_*(*t*) at the aortic root, as well as waves generated by LV contraction. Therefore, instead of defining a traditional, time-independent reflection coefficient, we introduced a time-varying emission coefficient, *γ*(*t*), accounting for waves generated (or “emitted”) by LV contraction. For any time in the cardiac cycle *γ*(*t*) is defined as

(2) $$\gamma =\frac{P_{f}}{P_{b}}.$$

This ratio quantifies the amount of pressure ‘emitted’ at the aortic root towards downstream vessels relative to the amount of pressure reaching the aortic root from downstream vessels. As such, *γ*(*t*) provides an instantaneous assessment of wave dynamics at the aortic root. The following derivation provides a hemodynamics explanation of why *γ*(*t*) calculated using Eq. (2) is approximately proportional to aortic blood flow, *Q*(*t*), as shown in Fig. 1(B) and observed in all three case studies.

Traditional wave separation analysis results in *P_f,b_* = ±*Z_c_*. *Q_f,b_* where *Z_c_* is the characteristic impedance, *Q_f_*(*t*) is the forward-traveling flow component, and *Q_b_*(*t*) is the backward-traveling flow component, so that the total flow is *Q = Q_f_ + Q_b_* [19]. Therefore, Eq. (2) becomes

(3) $$\gamma =\frac{Z_{c}Q_{f}}{P_{b}}=\frac{Z_{c}}{P_{b}}\left(Q-Q_{b}\right)=1+\frac{Z_{c}Q}{P_{b}}.$$

Moreover, $P_{b}(t)∼\frac{DBP}{2}$ in early systole (Fig. 1(A)) and, hence, during early systole *γ*(*t*) can be directly related to only one time-varying parameter that can be measured *in vivo*, *Q*(*t*), through the approximated expression

(4) $$\gamma ∼1+2\frac{Z_{c}\cdot Q}{DBP}.$$

Consequently, peak emission, *γ_peak_*, can be approximated as

(5) $$\gamma_{peak}∼1+2\frac{Z_{c}\cdot Q_{peak}}{DBP},$$

with *Q_peak_* the peak flow at the aortic root.

### C New Pressure Wave Separation

Our new wave separation analysis decomposes the CBPW into the five components described below.

#### 1) Microcirculation Pressure

In the absence of ventricular ejection, the CBPW would be entirely composed of the asymptotic pressure, *P_μ_*, at which flow to the microcirculation ceases and that we assumed to be constant.We calculated *P_μ_* by fitting a monoexponential curve of the form *P_μ_* + (*P_es_* – *P_μ_*)*exp(-*Bt*) to the diastolic part of *P*(*t*), with *P_es_* the end-systolic pressure and *P_μ_* and B the two parameters calculated from the fit. The pressure *P_μ_* forms the baseline of the net CBPW (Fig. 1(A), grey area).

#### 2) History Pressure

At the start of systole, before the aortic valve opens and the LV starts ejecting blood into the ascending aorta, the only waves present in the systemic circulation are history waves generated by previous cardiac contractions [10]. The history pressure component, *P_his_*(*t*), can be calculated by prolonging the diastolic decay in CBPW from the previous cardiac cycle into the current cycle (Fig. 1(A), green area) as described in [10]. Therefore, at the end of diastole and before the aortic valve opens, the net CBPW is made up entirely of the sum of the microcirculation and history pressure components.

#### 3) Cardiac-Aortic Coupling (Water-Hammer) Pressure

At the start of the cardiac cycle, the flow generated by LV contraction, *Q*(*t*), would be transformed into the water-hammer pressure, *P_wh_*(*t*), through the aortic impedance *Z_c_* as defined by *P_wh_* = *Z_c_* . *Q* [19] (Fig. 1(A), red area), in the absence of downstream reflected waves. This pressure component, therefore, originates from the coupling of LV ejection and the material properties of the ascending aorta that make up *Z_c_* = *ρc/A*, namely the luminal cross-sectional area, *A*, and pulse wave velocity, *c*. By taking *U = Q/A* we have

(6) Pwh=ρc U.

#### 4) Downstream Reflections Pressure

The pressure component generated by LV contraction within a cardiac cycle, *P_cc_*(*t*), is given by subtracting the microcirculation, *P_μ_*, and history, *P_his_*(*t*), pressure components from the net pressure wave, *P*(*t*); *i.e.*, *P_cc_* = *P* – *P_his_* – *P_μ_*. Traditional WSA applied to *P_cc_*(*t*) and *U*(*t*) via Eq. (1) allows the backward-traveling wave, *P_cc,b_*(*t*), to be calculated (Fig. 1(A), blue area):

(7) P cc,b=P down=12∑(dPcc−ρcdU).

We use the nomenclature *P_down_*(*t*) for this pressure component made up of all reflections originating downstream of the aortic root within the cardiac cycle being analyzed (Fig. 1(A), blue area). These reflections originate at proximal locations (*e.g*., due to aortic tapering) and at more distal locations due to peripheral reflections. It is important to note that the flow velocity wave driven by the pressure gradient generated by *P_cc_*(*t*) is *U*(*t*); *i.e.*, the same as the flow velocity wave driven by the pressure gradient produced by *P*(*t*). This is because the flow driven by the diastolic pressure decay at the aortic root is zero, under the assumption of zero blood flow during diastole, as described in [22]. As a result, traditional WSA can be applied to the pair *P_cc_*(*t*) and *U*(*t*) as done in Eq. (7).

#### 5) Aortic Re-Reflections Pressure

Traditional WSA applied to *P_cc_*(*t*) and *U*(*t*) via Eq. (1) produces the forward-traveling pressure component, *P_cc, f_*(*t*),

(8) $$P_{cc,f}=\frac{1}{2}\sum \left(dP_{cc}-\rho cdU\right).$$

*P_cc,f_* is composed of waves generated by LV contraction (*i.e.*, *P_wh_*) and re-reflections of *P_down_* at the aortic root, which we denote as *P_Ao_*(*t*); *i.e.*,

(9) $$P_{Ao}=P_{cc,f}-P_{wh}.$$

Supplemental Material S2 shows that *P_Ao_* must have the same magnitude as *P_down_*.

## IV Results

### A Similarity between Emission Coefficient and Aortic Flow

**Fig. 2:**
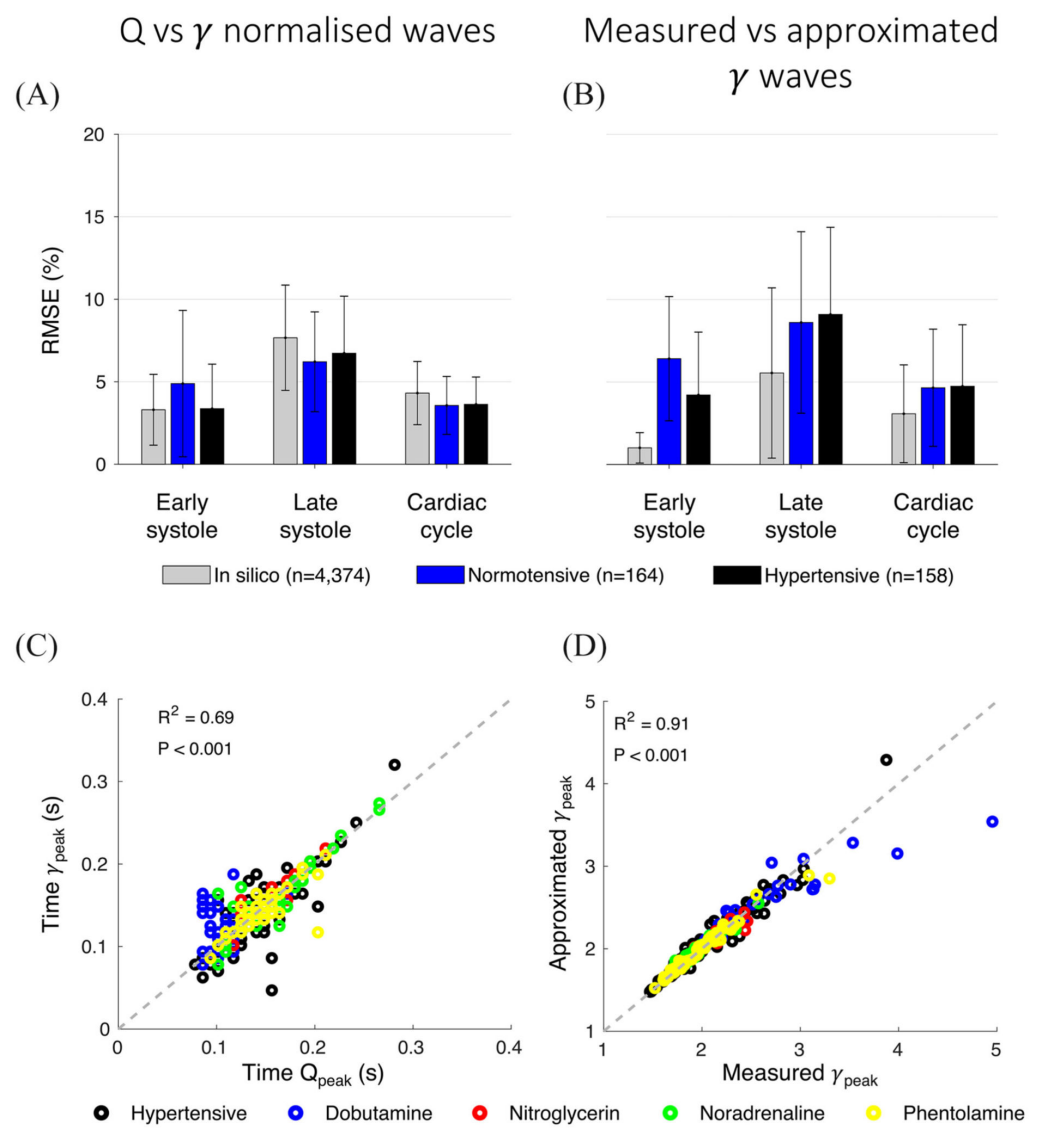
Relation of emission coefficient, *γ*(*t*), to aortic flow, *Q*(*t*), waves. (A) Relative root-mean-square error (RMSE) between normalized *γ* and *Q* for three different time spans (early systole until peak flow, late systole from peak flow to valve closure, and whole cardiac cycle) in the *in silico, in vivo* normotensive, and *in vivo* hypertensive groups. (B) Relative RMSE between measured and approximated *γ* calculated using Eqs. (2) and (4), respectively. (C) Scatter plot for the *in vivo* groups showing the direct relationship between the times of peak aortic *γ*, *γ_peak_*, and peak aortic flow, *Q_peak_* (coefficient of determination, R^2^=0.69). (D) Scatter plot for the *in vivo* groups comparing measured and approximated *γ_peak_*, the latter calculated by Eq. (5) (R^2^=0.91).

The patterns of the aortic flow wave *Q* and emission co-efficient *γ* over time are very similar throughout the cardiac cycle (Fig. 1(B)). When both waves were normalized by their peak values, relative root-mean-square errors (RMSEs) between *Q* and *γ* were consistent across the three groups of data considered (*in silico*, *in vivo* normotensive, and *in vivo* hypertensive): the relative RMSEs were <5% in early systole (until peak flow rate) and over the whole cardiac cycle; and between 5% and 10% during late systole (Fig. 2(A)). The timings of the respective peak values were highly correlated in the *in vivo* groups with a coefficient of determination R^2^ = 0.69 (Fig. 2(C)).

### B Comparison between Measured and Approximated Emission Coefficients

For all three data groups, comparison of the measured and approximated *γ*(*t*) patterns over time – calculated using Eqs. (2) and (4), respectively – led to relative RMSEs <5% over the whole cardiac cycle, with relative RMSEs smaller in early systole than in late systole (Fig. 2(B)). In the *in vivo* groups, the times of the measured and approximated *γ_peak_* were highly correlated (R^2^ =0.69, Supplemental Material Fig. S3(A)), while the magnitude of the measured *γ_peak_* was well described by Eq. (5) (R^2^ =0.91, Fig. 2(D)). An even stronger correlation between computed and approximated *γ_peak_* was observed in the *in silico* group (R^2^ = 0.96, Supplemental Material Fig. S3(B)).

### C Case Study 1: CBPW Components With Increasing Pulse Pressure

**Fig. 3:**
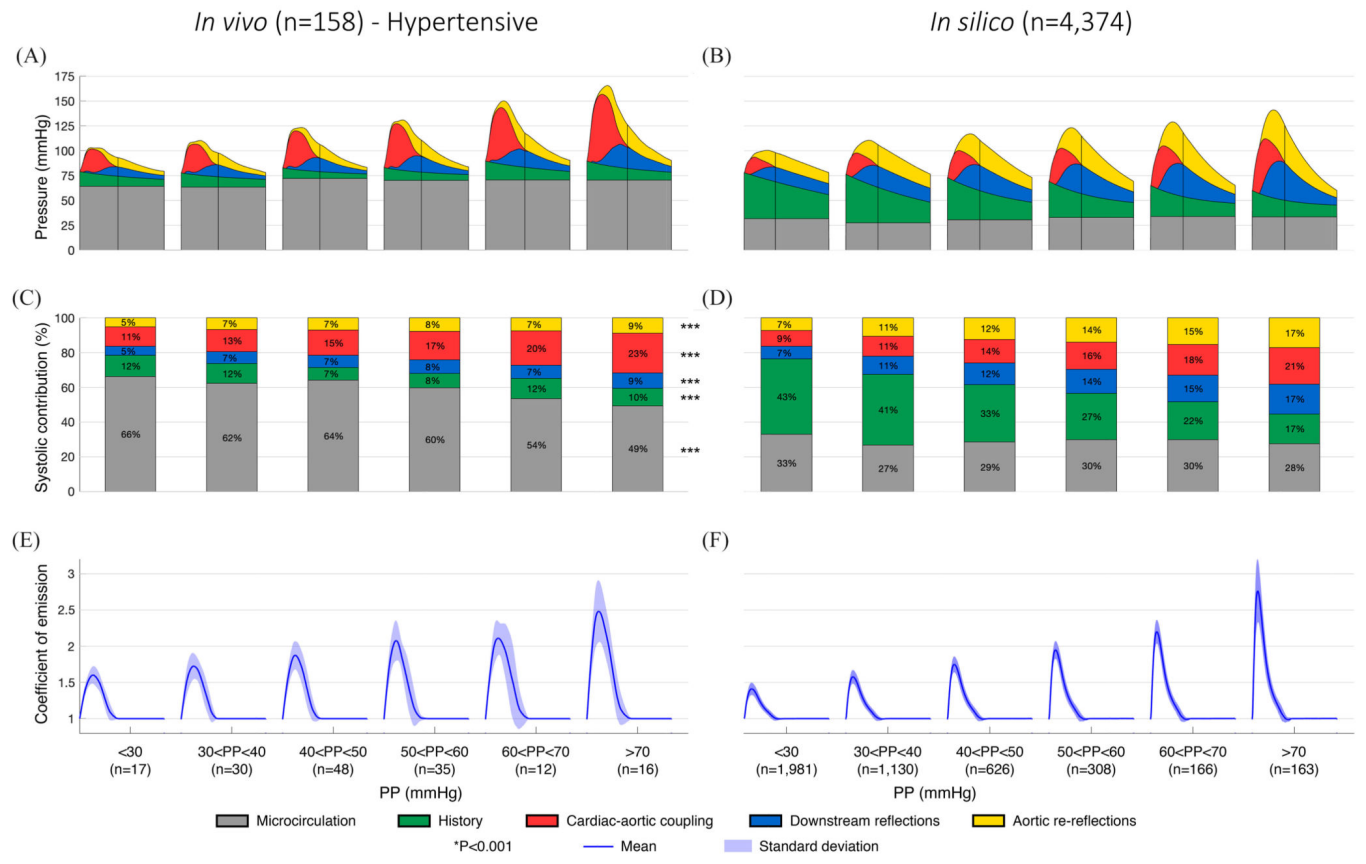
Variation of central pressure components and emission coefficient *γ* across 10-mmHg spans of pulse pressure (PP) in the *in vivo* hypertensive (left) and *in silico* (right) groups. (A, B) Pressure wave components obtained by the new WSA. (C, D) Relative contribution of each pressure component up to valve closure (straight vertical lines) expressed as a percentage of the area under the pressure wave during systole. (E, F) Mean emission coefficients (solid lines) and their standard deviations (shaded areas) calculated using Eq (2).

The relative contributions to the CBPW of the different pressure components obtained by the new WSA up to valve closure (straight vertical lines in Fig. 3(A) and B) were investigated across 10-mmHg spans of pulse pressure (PP) through comparison of areas under the pressure curve (Fig. 3(C) and (D)) for the hypertensive and *in silico* groups. The combined contribution of waves already present in the cardiovascular system at the start of the cardiac cycle being analyzed (*i.e*., microcirculation pressure, *P_μ_*, grey areas, and history pressure, *P_his_*, green areas) diminished in both groups with increasing PP (from PP < 30 mmHg to PP > 70 mmHg): from 78% to 59% in the hypertensive group (P<0.001, Fig. 3(C)) and from 76% to 45% in the *in silico* group (P < 0.001, Fig. 3(D)). The cardiac-aortic coupling (*P_wh_*, red areas), downstream reflections (*P_down_*, blue areas), and aortic re-reflections (*P_Ao_*, yellow areas) pressure components increased in both groups, with *P_wh_* showing a more predominant increase compared to *P_per_* and *P_Ao_*, especially in the hypertensive group (12% increase for *P_wh_* vs 4% for both *P_Ao_* and *P_down_*; P<0.001). These results indicate that *P_wh_* was primarily responsible for the increase in the magnitude of CBPW during systole with increasing PP, with pressure components due to peripheral reflections and aortic re-reflections having a secondary effect. Mean peak *γ* also increased with increasing PP: from 1.6 to 2.5 in the hypertensive group (P < 0.001, Fig. 3(E)) and from 1.4 to 2.8 in the *in silico* group (P < 0.001, Fig. 3(F)), suggesting that the cardiac-aortic coupling pressure component and wave reflections at the aortic root contribute significantly to the increase in PP.

### D Case Study 2: Impact of Enhanced Myocardial Contractility

**Fig. 4:**
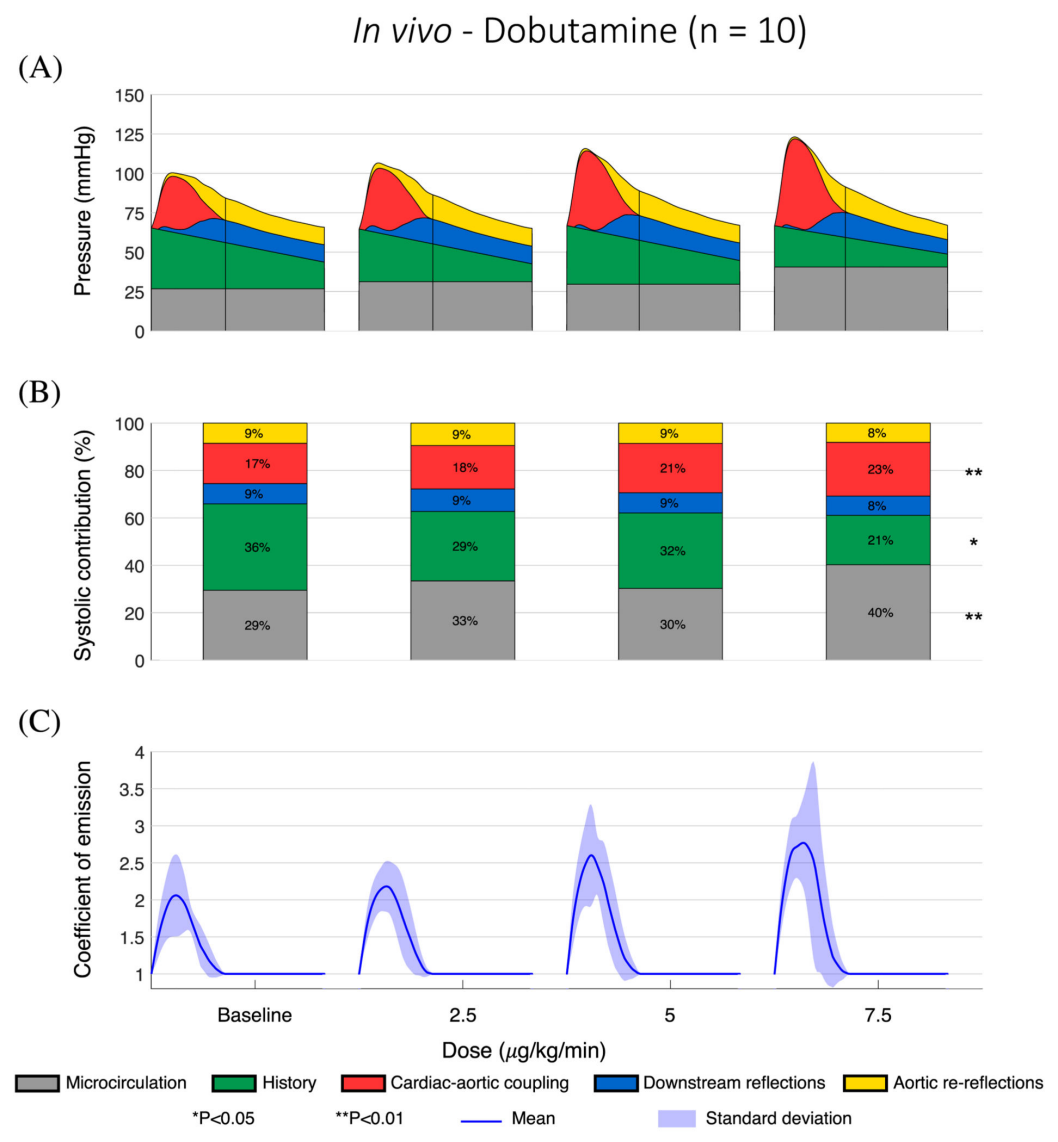
Variation of central pressure components and emission coefficient *γ* in a subset of the *in vivo* normotensive group (n = 10) with increasing doses of dobutamine, with the same format as Fig. 3.

PP increased with increasing dobutamine dose, from a mean of 35.0 mmHg at baseline to a mean of 56.4 mmHg for the highest dose (Fig. 4(A)). Only the change in the *P_wh_* component was statistically significant (P < 0.01) out of the three components arising during the cardiac cycle being analyzed, with *P_wh_* increasing from 17% to 23% (Fig. 4(B)). The contributions of *P_Ao_* and *P_down_* remained at around 9% (P = 0.18 for both). The combined contributions of *P_μ_* and *P_his_* remained at around 60-65% but they followed opposite trends: *P_μ_* increased from 29% to 40% (P < 0.01) while *P_his_* fell from 36% to 21% (P < 0.05). Peak *γ* increased from 2.1 to 2.7 (P < 0.05, Fig. 4(C)). These results indicate that changes in myocardial contractility led to variations in the (i) cardiac-aortic coupling pressure component and (ii) emission coefficient, suggesting that our novel WSA can describe the impact of cardiac function on CBPW.

### E Case Study 3: Suppression of Peripheral Wave Reflections

**Fig. 5:**
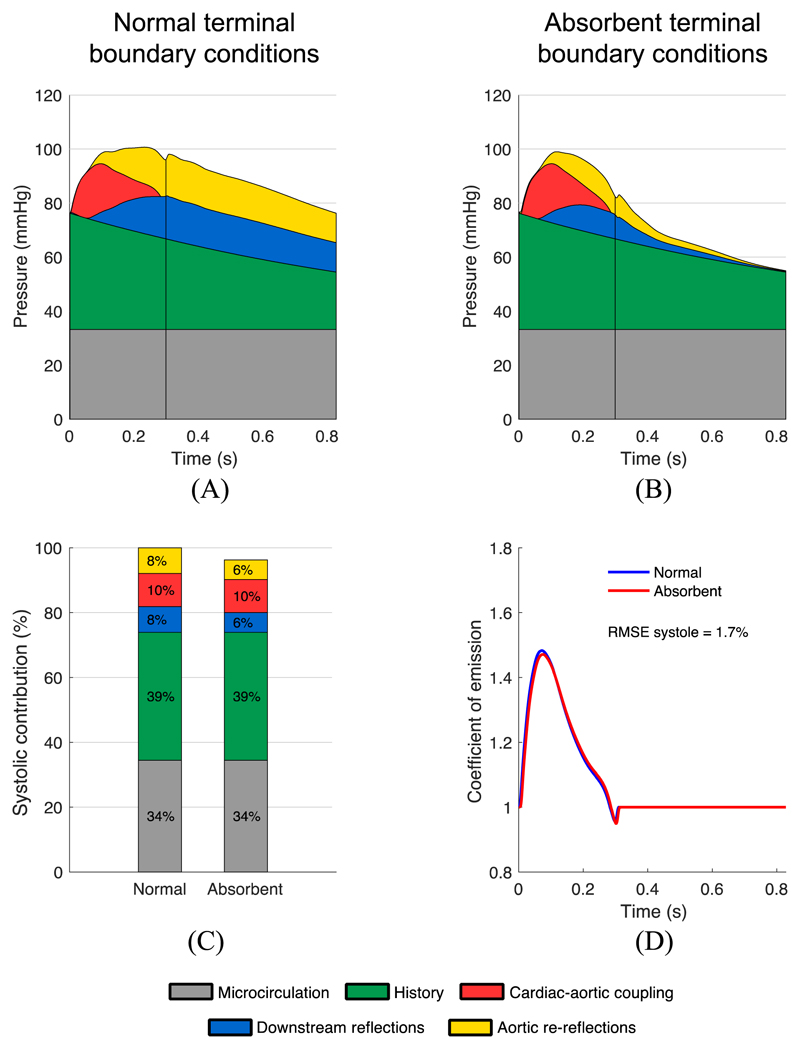
Effect of peripheral reflections on central pressure components and emission coefficient *γ*(*t*) for the 25 y.o. baseline subject of the *in silico* group. (A, B) Pressure wave components obtained by the new WSA applied to the baseline subject with normal (A) and fully absorbent (B) terminal boundary conditions (BCs). (C) Relative contribution of each pressure component up to valve closure (straight vertical lines) for the normal and absorbent cases, expressed as a percentage of the area under the pressure wave during systole of the model with normal terminal BCs. (D) Emission coefficient calculated using Eq. (2) with normal (blue) and fully absorbent (red) terminal BCs, with the relative root man square error (RMSE) between the two curves in systole shown in the legend.

For the baseline models of each age decade of the *in silico* group (from 25 to 75 y.o.), we simulated a scenario in which all terminal boundary conditions (BCs) were switched at the end of the previous cardiac cycle from normal (Fig. 5(A)) to fully absorbent (Fig. 5(B)). In the latter configuration, no reflections can come back from the most peripheral arteries to the aortic root and, hence, reflections arriving at the aortic root are entirely produced by sites of impedance mismatch within the arterial network; *i.e.*, due to tapering and arterial bifurcations [8]. Overall, the amplitudes of *P_down_* and *P_Ao_* had a greater increase across age decades when normal rather than absorbent BCs were used (*P_down_*: +10.7 mmHg vs +4.6 mmHg; *P_Ao_*: +8.5 mmHg vs +5.1 mmHg). By switching BCs to fully absorbent, the area under the systolic portion of the net pressure curve decreased by 4% in the 25 y.o. baseline model and 10% in the 75 y.o. baseline model; the difference being entirely due to a drop in the downstream reflections and aortic re-reflections components of pressure, in equal proportions (Fig. 5(C)). In contrast, the BC switch had very little effect on the variations of *γ* over time (Fig. 5(D)). As a result, the relative RMSE for *γ* in systole between the two BC types was between 1.7% and 3.1% for any age group, with a mean difference for peak *γ* of 0.7%. These results suggest that the emission coefficient is not influenced by peripheral wave reflections. Fig. 5 shows the results for the 25 y.o. baseline subject and Supplemental Materials Fig. S4 contains the results for the 35, 45, 55, 65 and 75 y.o. baseline subjects.

## V Discussion

Understanding the hemodynamic determinants of the central blood pressure wave (CBPW) is key to targeting appropriate treatment strategies that prevent and treat the large burden of cardiovascular disease associated with hypertension in middle-aged to older persons. We have introduced a new physics-based decomposition of the CBPW into five hemodynamic components and demonstrated its ability to identify and quantify the biophysical location and temporal origin of each component. The new decomposition is based on the same inputs as traditional WSA (pressure and flow waves, and aortic impedance). It separates microcirculation (*P_μ_*) and history (*P_his_*) pressure components originated in previous cardiac cycles from three additional pressure components arising specifically as a result of ventricular contraction at the start of the cardiac cycle being analyzed: (i) a component generated by ventricular-aortic coupling (*P_wh_*) accounting for the interaction between the blood volume ejected during cardiac contraction and the luminal area and wall stiffness of the ascending aorta through the aortic characteristic impedance; (ii) a component accounting for the amount of downstream reflections from bifurcations, tapering and distal blood vessels (*P_down_*); and (iii) a component (*P_Ao_*) made up of re-reflected waves of the aforementioned *P_down_* at the aortic root. *P_wh_* and *P_Ao_* waves travel from the aortic valve to the periphery, the latter resulting from aortic reflections of *P_down_* waves. Key to our novel CBPW decomposition is the introduction of a time-varying emission coefficient (*γ*) that quantifies pulse wave reflections at the aortic root, showing that the aortic root is a major reflection site in the systemic circulation. In addition, this is the first time, to our knowledge, that the impact of microcirculation and history pressure components on the net pressure wave is studied *in vivo.*

This study has identified the cardiac-aortic coupling pressure component as the most important contributor to the increase in CBPW during systole – including central pulse pressure – out of the three components produced by ventricular ejection during the cardiac cycle being analyzed. It has, therefore, highlighted the main role played by the physical determinants of *P_wh_* (aortic flow, aortic luminal area, and aortic stiffness) in CBPW morphology. The importance of aortic flow in increasing central pulse pressure with hypertension has also being underlined through *γ*.

Our results were obtained by using a complementary mix of *in silico* and *in vivo* data: *in silico* simulations allowed us to test theoretical predictions in the absence of experimental errors and peripheral wave reflections, while *in vivo* data obtained in hypertensive patients and volunteers whose hemodynamics properties were pharmacologically altered further strengthened *in silico* observations without resorting to modeling hypotheses.

### A Hemodynamic Genesis of Reflected Waves

Our novel WSA differentiates between traditional backward-traveling reflections from downstream to the aortic root and forward re-reflections generated at the aortic root. Historically, only backward reflections were investigated since from a physiological standpoint they were seen as the major determinant in the augmentation of CBPW above the first systolic shoulder up to peak pressure; a feature that has been demonstrated to disproportionally contribute to the age-induced elevation in blood pressure [23] and, when expressed as a quotient of central pulse pressure in the form of the augmentation index (AIx), has been associated with clinical events independently from central pulse pressure [24].

Early works theorized the locations of a few main reflection sites away from the aortic root [7], [8], though the current general consensus is that reflections arise from a myriad of sites in the systemic arterial network as forward waves encounter sites of impedance mismatch such as tapered vessels and bifurcations [6]. In this study, we have shown that there is a theoretical case to consider the aortic root as a major reflection site of the systemic circulation. Forward waves are generated at the aortic root because of LV contraction and re-reflection of backward waves arriving at the aortic root from the downstream vasculature, including distal locations. Therefore, forward waves at the aortic root are influenced by proximal aortic properties and aortic flow through the cardiac-aortic coupling pressure, *P_wh_*, as well as by distal arterial properties through the aortic re-reflections pressure, *P_Ao_*, in agreement with Phan *et al.* [11]. Moreover, given the similarity in morphology between the emission coefficient, *γ*(*t*), and the aortic blood flow, *Q*(*t*), the latter is a very good approximation of how the aortic root transforms backward waves into forward waves throughout the cardiac cycle. The similarity between *γ* and aortic flow over a cardiac period (Fig. 1(B)) is supported by both our new theory – as indicated by Eqs. (3) to (5) – and the experiments conducted with a complementary mix of *in silico* and *in vivo* data.

At the start of the next cardiac cycle, *P_down_* and *P_Ao_* from the previous cycle become part of the history pressure wave of the new cardiac cycle, so that the left ventricle needs to generate only a portion of the aortic pressure. The history pressure can be subdivided into a series of components containing reflected and re-reflected waves arising from several prior beats and decaying at a rate proportional to the diastolic time constant of the arterial system [10].

### B Differences With The Reservoir-Wave Separation Method

Fundamental differences exist between our novel WSA and the reservoir-wave analysis (RWA). Both methods have a component proportional to the flow (the excess pressure in the RWA, the water-hammer pressure in our WSA). However, the excess pressure is dependent upon reservoir characteristics, since it is defined as the remaining pressure after subtraction of the reservoir pressure from the total pressure [25], while the water-hammer pressure is calculated from the flow, independently from the total pressure (and hence reservoir characteristics). This is in agreement with the observation that the pulsatile CBPW, in the absence of reflections and during early systole, can be entirely captured by the water-hammer pressure [26]. Both analyses have a Windkessel-type pressure component (the reservoir pressure in the RWA, the history pressure in ours), however the reservoir pressure is assumed to be space-independent [27] whereas the history pressure component is made up of traveling waves originating in previous cardiac cycles [10], avoiding fundamental hemodynamic concerns raised against the existence of a space-invariant, wave-independent reservoir pressure component [9], [28]. It should also be noted that the RWA is performed on the whole pressure wave [25] while we only considered the part of the pressure wave arising exclusively from cardiac contraction; *i.e.*, after subtraction of the microcirculation and history pressure components. Such subtraction has been shown to provide more accurate pulse wave velocity estimates in rabbits [22] and *in silico* [22], [29].

### C Similarities With the Wave-Potential Concept

That the sum of forward- and backward-traveling waves can behave like a Windkessel has been explained by Mynard and Smolich [20] by introducing a comprehensive wave-based explanation of arterial hemodynamics termed wave potential (WP). Briefly, assuming that wave reflections are distributive in nature and can be re-reflected, the multiple re-reflections of the late systolic forward-expansion pressure waves, along with the discharge of a “one-dimensional Windkessel” during diastole, produce self-cancelling waves and a quasi-exponential pressure decay. The wave trapping phenomenon that this entails has been previously described [5] and is also hinted at in our study, as pressure reflections from the most terminal vessels contribute little to the emission coefficient at the aortic root (Fig. 5(D)).

Another assumption of the WP concept is that the aortic valve constitutes a reflection site with a coefficient close to unity to explain the equal forward- and backward-traveling pressure waveforms but opposite forward- and backward-traveling flow waveforms during diastole. This is compatible with the time-varying emission coefficient *γ* that has been introduced in this study. *γ* can be understood as the addition of a traditional reflection coefficient that is close to unity throughout the cardiac cycle and an ejection coefficient that is proportional to the aortic flow, oscillating between values greater than zero during systole and zero in diastole. This results in forward-traveling waves being generated at the aortic root throughout the cardiac cycle with a total coefficient *γ* that is greater than one in systole and close to one in diastole. Backward-traveling waves arriving at the aortic root may affect ventricular outflow and, as a result, the amount of wave reflection at the aortic root may be less than one during systole [12].

### D Therapeutic Implications

From a therapeutic standpoint, our results reaffirm the preponderance of ventricular dynamics and aortic stiffness as the main mechanisms underlying the elevation of pulsatile blood pressure found in previous studies, both in hypertensive patients and in normotensive subjects in whom normal physiology was modulated over a large range by inotropic and vasoactive drugs [30]. Given the equality in magnitude between forward-travelling re-reflections and backward-travelling downstream reflections obtained from the new pressure wave separation, the rise in central blood pressure is dependent upon what triggers the backward reflections in the first place, *i.e.*, the original pressure wave generated by cardiac contraction. Most treatments for hypertension are thought to work by reducing peripheral vascular resistance and hence mean arterial blood pressure, with an indirect effect on conduit arteries resulting from pressure-dependence of arterial stiffness. The present findings highlight the potential importance of targeting ventricular-vascular coupling directly, either through a direct action on large arteries or on the heart.

Another major finding of this new decomposition is the decreasing importance of history waves as pulse pressure rises and diastolic exponential decay markedly falls, resulting in a greater contribution to CBPW by LV contraction within each cardiac cycle (Figs. 3(C), (D) and 4(B)). As a result, more physical work needs to be done by the LV to eject blood flow into the aorta, which may result in the ventricle to develop hypertrophy and fail. The time constant of the exponential decay is equal to the product between total arterial compliance and peripheral resistance and, hence, a greater decay can be explained by a decrease in compliance – associated with artery stiffening – leading to a smaller time constant. This indicates that strategies targeting aortic stiffness, hence increasing compliance of the most proximal vessels, might be effective at reducing both blood pressure and LV work by increasing the contribution to CBPW of history waves.

Finally, our results show that strategies aiming to reduce microcirculation pressure, for example by targeting capillary rarefaction [31] or decreasing venous pressure by reversing sodium and volume overload [32], could be an effective means to treat hypertension since the microcirculation pressure component contributed between 49% and 66% to the CBPW in the hypertensive cohort.

### E Limitations

The microcirculation and history pressures were calculated by fitting a monoexponential to the diastolic part of the pressure waveform. This approach may be unreliable in cases were a clear exponential decay is not present [33], and does not consider the pressure dependency of arterial compliance, which contributes to the time constant of the exponential fit.

Furthermore, we did not investigate how absorption/ attenuation in physical entities of the aortic root region (e.g., the ventricle, outflow tract, aortic valve, and coronary arteries), as well as their structural and functional alteration could affect the emission coefficient, *γ*, and, hence, the generation of forward waves at the aortic root. Fluid-structure interaction simulations could allow us to investigate the physical entities responsible for producing forward waves at the aortic root and understand their underlying mechanisms.

### F Perspectives

The origin of a main reflection site in the vascular tree has remained elusive for years despite reflected waves being attributed an important role in the elevation of pulsatile pressure. By introducing a new wave separation analysis and time-varying emission coefficient, we have shown that the aortic root is a major source of wave reflections in the vasculature and that the shape of the aortic flow wave is a good measure of how backward reflections are transformed into forward waves throughout the cardiac cycle. Therefore, therapeutic strategies aiming to treat hypertension should focus on the heart and proximal conduit arteries. Conditions and drugs that influence cardiac function and aortic stiffness may, therefore, influence central blood pressure wave morphology independent of peripheral vascular properties. Interventions to modulate ventricular dynamics might be useful in addition to reducing aortic stiffness in preventing/treating systolic hypertension.

## VI Conclusion

The aortic root is a major reflection site in the systemic arterial network and pulse wave dynamics at this location is the main driver of the increase in central blood pressure. Treatments targeting the ventricular-aortic interaction should be favored when tackling essential hypertension.

## Supplementary Material

Supplementary Material
