# Supplementary material for: Novel Pressure Wave Separation Analysis for Cardiovascular Function Assessment Highlights Major Role of Aortic Root
Source: IEEE Trans Biomed Eng. Author manuscript; Available in PMC 2022 Jun 30. (PMC7612937; doi:10.1109/TBME.2021.3127799)
Supplement: Supplementary Material [file EMS146313-supplement-Supplementary_Material.pdf]

Supplemental Material

Novel pressure wave separation analysis for cardiovascular function assessment highlights major role of aortic root

Samuel Vennin[1,2], Ye Li[1], Jorge Mariscal-Harana[2], Peter H. Charlton[2], Henry Fok[1], Haotian Gu[1], Phil Chowienczyk[1], Jordi Alastruey[2]

[1]King's College London British Heart Foundation Centre, Department of Clinical Pharmacology, St Thomas' Hospital, London, United Kingdom

[2]School of Biomedical Engineering and Imaging Sciences, King's College London, St. Thomas' Hospital, London, United Kingdom

Running title: Novel pressure wave separation analysis

Correspondence

Dr Jordi Alastruey, School of Biomedical Engineering and Imaging Sciences, King's College London, St. Thomas' Hospital, London, United Kingdom

E-mail: jordi.alastruey-arimon@kcl.ac.uk

S1 Subject characteristics

S1.1 Characteristics of the hypertensive group

Characteristics	Mean±SD or n
Age, yr	46.2±17.1
Sex, male, %	53
BMI, kg/m^2	26.9±4.4
**Drug therapy**	
ACEI, %	15
ARB, %	8
β-Blocker, %	12
Calcium channel blocker, %	23
Diuretic, %	7
α-Blocker, %	6
**Flow and pressure waveform**	
HR, bpm	65.4±17.5
pSBP, mmHg	138.2±26.2
DBP, mmHg	82.8±21.2
cSBP, mmHg	129.5±27.5
cPP, mmHg	47.0±13.7
AP, mmHg	4.0±16.2
AIx, %	6.7±2.6
PWV, m/s	5.2±2.4
Umax, m/s	1.1±0.4

Table S1: Detailed characteristics of the hypertensive group. ACEI, angiotensin-converting enzyme inhibitor; AIx, augmentation index; AP, augmentation pressure; ARB, angiotensin receptor blocker; BMI, body mass index; cPP, central pulse pressure; cSBP, central systolic blood pressure; DBP, diastolic blood pressure; HR, heart rate; pSBP, peripheral systolic blood pressure; PWV, pulse wave velocity; and Umax maximum flow velocity. Results are presented as mean±SD or as a percentage. Table adapted from [1].

S1.2 Characteristics of the normotensive group

Dose	HR (bpm)	DBP (mmHg)	P1 (mmHg)	P2 (mmHg)	PWV (m/s)	Umax (m/s)
			**Dobutamine (μg/kg/min)**			
**baseline**	64.7±3.5	65.4±3.1	36.8±3.7	32.5±3.0	4.1±0.4	1.16±0.07
**2.5**	66.7±4.5	64.7±2.8	43.7±3.2	38.0±2.3	4.5±0.4	1.32±0.05
**5**	69.0±4.7	66.8±2.3	51.3±3.8	41.4±2.5	5.1±0.4	1.37±0.04
**7.5**	73.0±5.2	66.8±2.0	59.0±3.4	47.2±3.2	5.6±0.3	1.42±0.04
**P value**	**0.024**	**0.58**	**<0.001**	**0.002**	**0.003**	**0.007**
			**Norepinephrine (ng/kg/min)**			
**Baseline**	60.9±3.2	66.2±3.1	35.3±1.7	33.0±3.0	4.4±0.3	1.14±0.03
**12.5**	56.5±3.2	71.1±3.0	36.5±2.0	35.8±2.3	4.7±0.4	1.15±0.04
**25**	54.3±3.1	74.2±3.0	33.1±1.4	37.3±2.7	4.7±0.3	1.07±0.05
**50**	52.3±2.9	78.7±2.9	36.8±2.8	43.7±4.3	5.6±0.6	1.07±0.06
**P value**	**<0.001**	**<0.001**	**0.25**	**0.001**	**0.11**	**0.24**
			**Phentolamine (μg/min)***			
**Baseline**	61.9±1.7	75.7±3.0	30.4±1.8	31.8±3.1	4.4±0.4	1.03±0.04
**25**	61.3±2.4	72.2±3.5	32.1±3.0	34.0±3.5	4.5±0.4	1.11±0.04
**50**	62.8±2.7	71.0±4.1	32.8±2.0	32.5±2.8	4.1±0.3	1.22±0.05
**100**	62.6±2.3	70.2±3.6	36.1±3.6	33.7±4.1	4.8±0.4	1.16±0.07
**P value**	**0.39**	**0.021**	**0.13**	**0.42**	**0.41**	**0.098**
			**Nitroglycerine (μg/min)**			
**Baseline**	63.4±2.7	69.4±3.4	36.6±3.0	36.0±3.9	5.2±0.6	1.09±0.06
**3**	59.8±2.8	64.9±3.3	34.8±2.2	32.4±4.3	4.8±0.3	1.10±0.04
**10**	61.2±2.5	65.0±3.3	35.1±2.4	30.4±4.2	5.1±0.5	1.08±0.05
**30**	62.1±2.5	62.5±3.9	35.8±2.7	29.6±4.9	5.2±0.4	1.07±0.03
**P value**	**0.26**	**0.018**	**0.70**	**0.033**	**0.63**	**0.79**

Table S2: Detailed characteristics of the normotensive group. HR, heart rate; DBP, diastolic blood pressure; P1, height above DBP of the shoulder of aortic pressure; P2, height above DBP of the second systolic shoulder of aortic pressure; PWV, pulse wave velocity; Umax, maximum flow velocity. *With preceding boluses of 1, 2 and 4 mg for infusions of 25, 50 and 100 μg/min respectively.

S2 Aortic re-reflection pressure ($\boldsymbol{P_{Ao}}$) and peripheral reflections pressure ($\boldsymbol{P_{per}}$)

Section C, 5) in Methods shows that the forward-travelling component, $P_{cc,f}(t)$, of the pressure generated by LV contraction within a cardiac cycle, $P_{cc}(t)$, can be expressed as $P_{cc,f} = P_{wh} + P_{Ao}$, where $P_{wh}(t) = Z_c \cdot Q$ is the water hammer pressure as defined by Parker [2] and $P_{Ao}(t)$ is the aortic re-reflections pressure component. By applying the method of characteristics [3] to $P_{cc}(t)$ and $Q(t)$ we can show that P_{Ao} has the same magnitude as the peripheral reflections pressure component, $P_{down}(t)$. According to this method, P_{cc} can be separated into forward, $P_{cc,f}(t)$, and backward, $P_{cc,b}(t)$, travelling components by introducing the linear forward and backward Riemann variables, $W_{f,b}(t) = Q \pm \frac{P_{cc}}{Z_c}$ [4]. In the forward-travelling direction we have

$$P_{cc,f} = Z_c \frac{W_f}{2}, \tag{1}$$

$$W_f = Q + \frac{P_{cc}}{Z_c}. \tag{2}$$

Substitution of W_f into $P_{cc,f}$ yields

$$P_{cc,f} = \frac{1}{2}[P_{cc} + Z_c Q]. \tag{3}$$

Subtraction of $P_{wh} = Z_c \cdot Q$ from Eq. (3) leads to

$$P_{cc,f} - P_{wh} = \frac{1}{2}[P_{cc} - Z_c Q]. \tag{4}$$

Similarly, in the backward-traveling direction we have

$$P_{cc,b} = -Z_c \frac{W_b}{2}, \tag{5}$$

$$W_b = Q - \frac{P_{cc}}{Z_c}, \tag{6}$$

which combine to produce

$$P_{cc,b} = \frac{1}{2}[P_{cc} - Z_c Q]. \tag{7}$$

As described in Section C, 4) in Methods, $P_{cc,b}$ is the peripheral reflections pressure made up of all peripheral reflections originating downstream of the aortic root within the cardiac cycle being analyzed; *i.e.* $P_{cc,b} = P_{down}$. As a result, combining Eqs. (4) and (7) leads to

$$P_{cc,f} = P_{wh} + P_{down} \quad (8)$$

and, hence,

$$P_{down} = P_{cc,f} - P_{wh}. \quad (9)$$

Comparing this equation with Eq. (9) in the main text we have that P_{Ao} must have the same magnitude as P_{down}.

S3 Comparison between measured and approximated emission coefficients – Additional figures

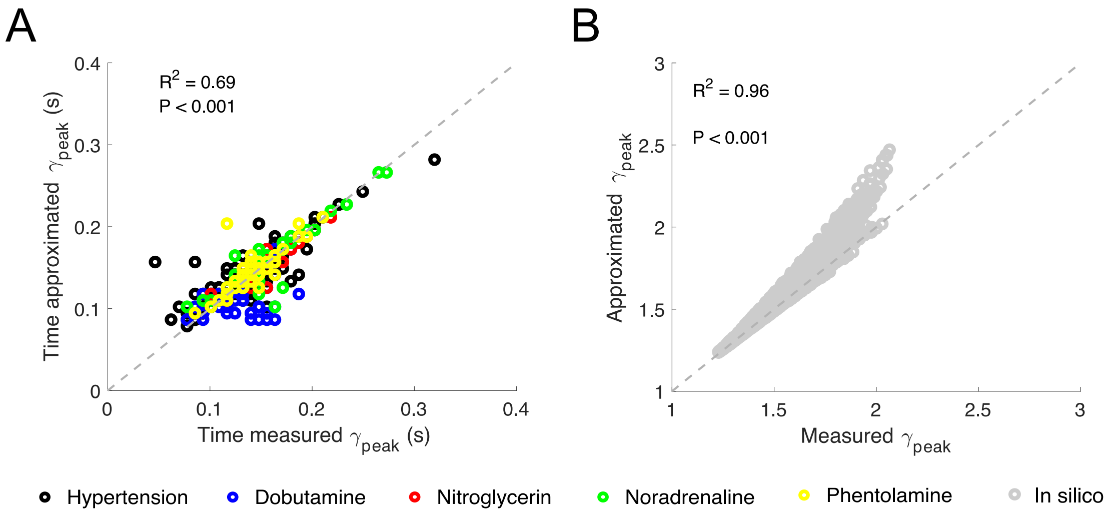

Figure S3. Comparison between properties of the measured and approximated emission coefficient, $\gamma(t)$, calculated from Eqs. (2) and (4), respectively, in the main article. In the *in vivo* group, the time at which peak emission, γ_{peak}, was reached was similar in the measured and approximated $\gamma(t)$ (R^2=0.69, panel A), while the magnitudes of measured and approximated γ_{peak} in the *in silico* group were also highly correlated (R^2=0.96, panel B).

S4 Suppression of peripheral wave reflections – Additional figures

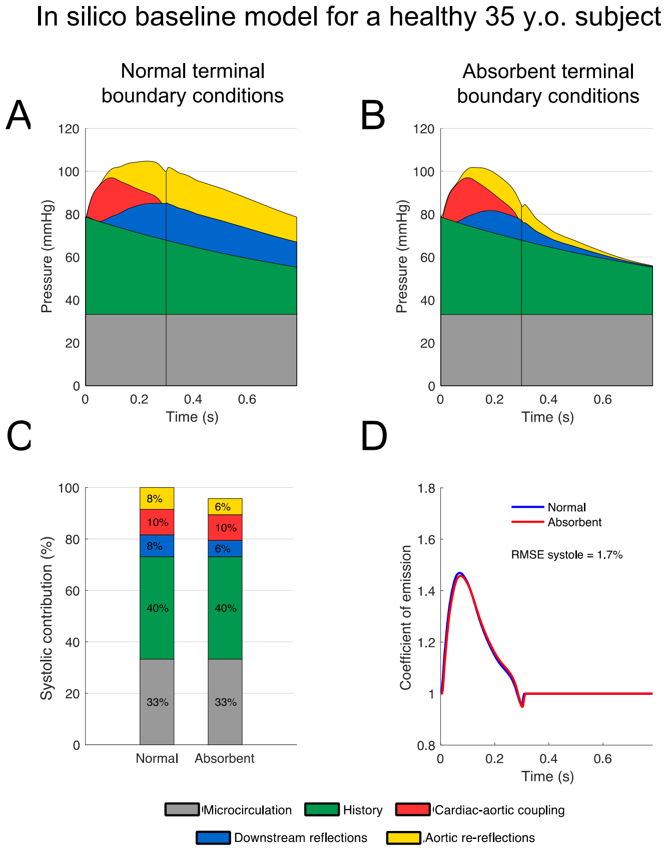

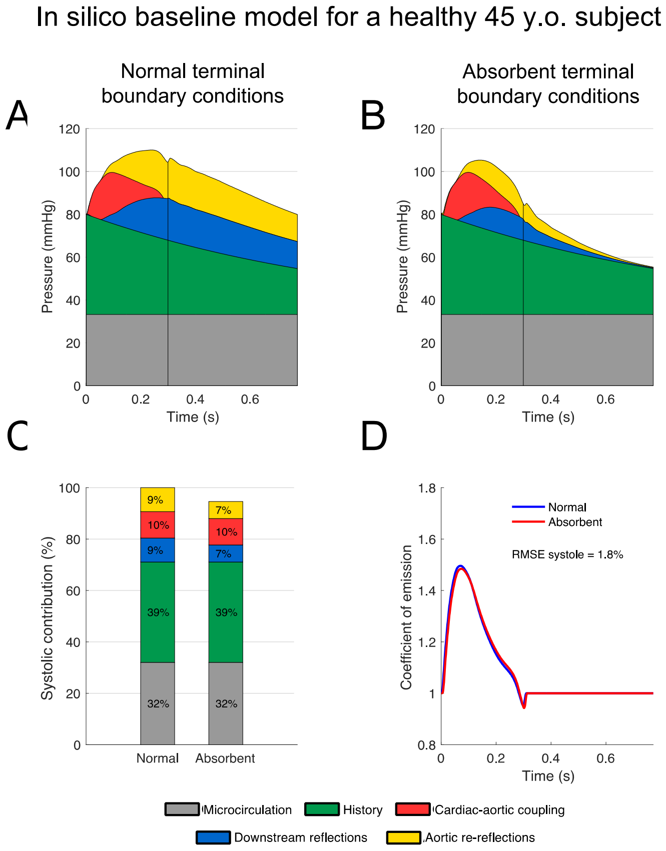

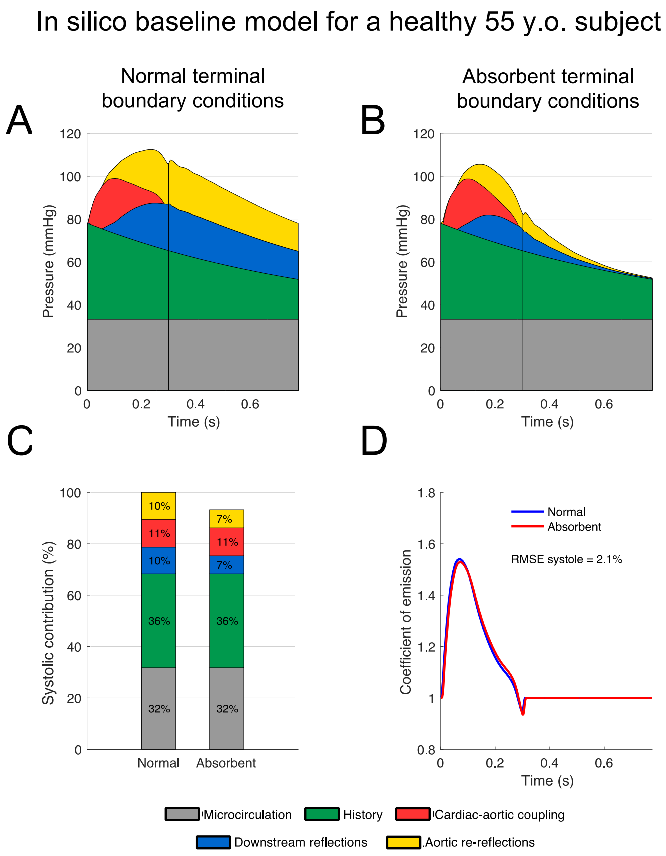
In silico baseline model for a healthy 55 y.o. subject
A
Normal terminal boundary conditions
B
Absorbent terminal boundary conditions
Pressure (mmHg)
Time (s)
C
Systolic contribution (%)
10%
11%
10%
36%
32%
7%
11%
7%
36%
32%
Normal
Absorbent
D
Coefficient of emission
Normal
Absorbent
RMSE systole = 2.1%
Time (s)
Microcirculation
History
Cardiac-aortic coupling
Downstream reflections
Aortic re-reflections

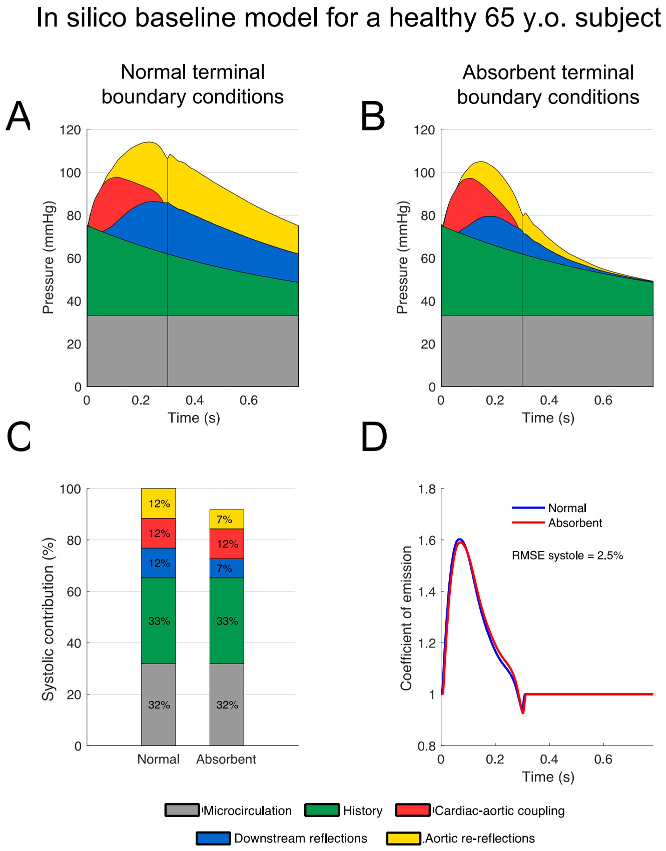
In silico baseline model for a healthy 65 y.o. subject
A
Normal terminal boundary conditions
B
Absorbent terminal boundary conditions
Pressure (mmHg)
Time (s)
C
Systolic contribution (%)
12%
12%
12%
33%
32%
7%
12%
7%
33%
32%
Normal
Absorbent
D
Coefficient of emission
Normal
Absorbent
RMSE systole = 2.5%
Time (s)
Microcirculation
History
Cardiac-aortic coupling
Downstream reflections
Aortic re-reflections

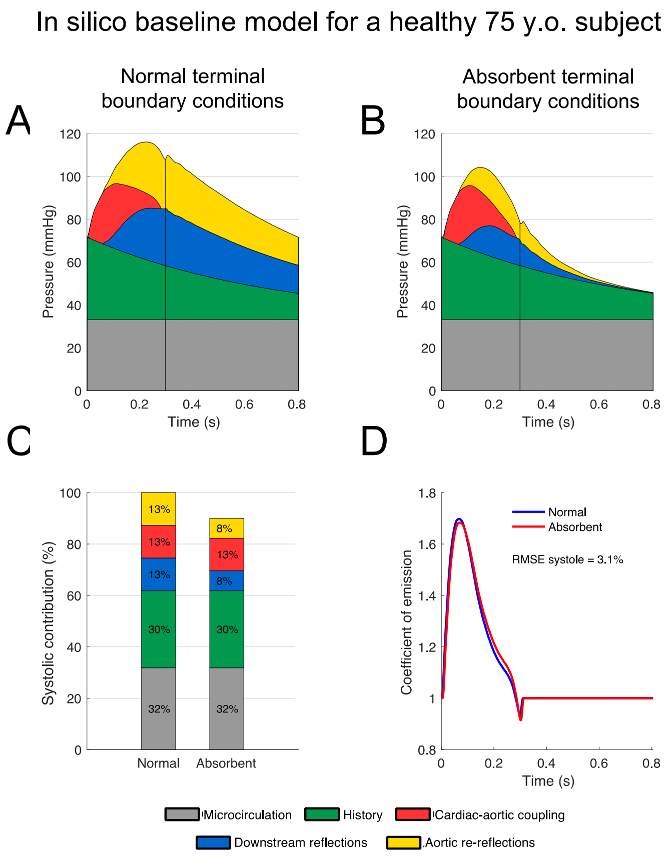

Figures S4. Effect of peripheral reflections on central pressure components and emission coefficient $\gamma(t)$ for the 35, 45, 55, 65 and 75 y.o. baseline subjects of the *in silico* group, with the same format as Fig. 5 showing the results for the 25 y.o. baseline subject.

Reference

[1] Y. Li, H. Gu, H. Fok, J. Alastruey, and P. Chowienczyk, “Forward and Backward Pressure Waveform Morphology in Hypertension,” *Hypertension*, vol. 69, no. 2, pp. 375–381, 2017, doi: 10.1161/HYPERTENSIONAHA.116.08089.

[2] K. H. Parker, “An introduction to wave intensity analysis.,” *Med. Biol. Eng. Comput.*, vol. 47, no. 2, pp. 175–88, Mar. 2009, doi: 10.1007/s11517-009-0439-y.

[3] J. Alastruey, K. H. Parker, and S. J. Sherwin, “Arterial pulse wave haemodynamics,” in 11th International Conference on Pressure Surges, pp. 401–442, Virtual PiE Led t/a BHR Group: Lisbon, Portugal, 2012.

[4] J. Alastruey, K. H. Parker, J. Peiró, and S. J. Sherwin, “Analysing the pattern of pulse waves in arterial networks: A time-domain study,” *J. Eng. Math.*, vol. 64, no. 4, pp. 331–351, 2009, doi: 10.1007/s10665-009-9275-1.
